# At seeming safe concentrations, synergistic effects of PM_2.5_ and formaldehyde co-exposure induces Alzheimer-like changes in mouse brain

**DOI:** 10.18632/oncotarget.21637

**Published:** 2017-10-06

**Authors:** Xudong Liu, Yuchao Zhang, Chen Luo, Jun Kang, Jinquan Li, Kun Wang, Ping Ma, Xu Yang

**Affiliations:** ^1^ Laboratory of Environmental Biomedicine, Hubei Key Laboratory of Genetic Regulation and Integrative Biology, College of Life Science, Central China Normal University, Wuhan, China; ^2^ Department of Food science and Engineering, Moutai College, Renhuai, China; ^3^ Research Center of Basic Medical Sciences, School of Basic Medical Sciences, Hubei University of Science and Technology, Xianning, China; ^4^ New York University School of Medicine, Tuxedo, New York, United States of America

**Keywords:** Alzheimer’s disease, air pollution, co-exposure, oxidative stress, inflammation

## Abstract

Alzheimer's disease (AD) is a serious, common, global disease, yet its etiology and pathogenesis are incompletely understood. Air pollution is a multi-pollutants co-exposure system, which may affect brain. The indoor environment is where exposure to both air particulate matter (<2.5 μm in diameter) (PM_2.5_) and formaldehyde (FA) can occur simultaneously. Whether exposure to such a multi-pollutant (PM_2.5_ plus FA) mixture contributes to the development of AD, and whether there is a difference between exposure to PM_2.5_ or FA alone needs to be investigated. To determine the objective, C57BL/6J mice were exposed daily to PM_2.5_ (0.193 mg/Kg/day), FA (0.155 mg/Kg/day) or multi-pullutants (0.193 mg/Kg/day PM_2.5_ plus 0.155 mg/Kg/day FA) for one week. AD-like changes and upstream events were investigated after exposure. The results showed that exposure to PM_2.5_ or FA alone in this study had little or no adverse effects on the mouse brain. However, some AD-like pathologies were detected after multi-pullutants co-exposure. This work suggested PM_2.5_ plus FA co-exposure has more potential to induce AD-like pathologies than exposure alone. Oxidative stress and inflammation may be involved into the toxic mechanisms. Synergistic effects of co-exposure may induce the hygienic or safety standards of each pollutant not safe.

## INTRODUCTION

Alzheimer's disease (AD) is a serious, common, global disease, yet its etiology and pathogenesis remain incompletely understood. Many factors have been reported to contribute to the etiology of AD, such as genetic factors, environmental factors and human lifestyle patterns [1–5]. Air pollution is a multifaceted environmental toxin, comprising particulate matter (PM), gases, organic compounds and metals, and is present in indoor and outdoor air [6]. Recently, increasing evidence suggests that exposure to air pollution not only harms the human heart and lungs, but it may also affect the brain [7].

Different countries or organizations have their own atmospheric environment safe, quality or hygienic standards. In our daily life, people usually consider that when we exposure to certain pollutant at its standard even slightly higher than the standard has zero or minor adverse effects. Actually, we have a mistake, the safe standard developed generally according to scientific researches which the pollutant exposure alone. In fact, environmental toxin exposure is a multi-pollutants co-exposure system, for example air pollution.

Air particulate matter (<2.5 μm in diameter) (PM_2.5_) and formaldehyde (FA) are considered as one of the most important outdoor and indoor air pollutant respectively. The co-exposure of them seems to be impossible, while outdoor PM_2.5_ can enter buildings by infiltration and ventilation. Add the indoor source PM_2.5_, the concentration of indoor PM_2.5_ tends to be the same as outdoor’s, and in some cases may be even higher [8]. Since people typically spend more than 90% of their time indoors [9], extended co-exposure to PM_2.5_ and FA at indoor is highly likely.

Although epidemiological studies have shown that there is a correlation between exposure to certain specific air pollutants and the occurrence of AD, evaluating the risk of AD after co-exposure to multi-pollutants has not been extensively studied. The aims of this study are: (1) identify whether there are differences in the development of AD due to exposure to PM_2.5_, FA or a multi-pollutant (PM_2.5_ plus FA); (2) explore the possible mechanisms of the effects.

## RESULTS

### Components found in PM_2.5_ samples

Table 1 shows the results of elements analysis. A total of elements were identified as well as element carbon (EC) and organic carbon (OC). Table 2 shows concentrations of water soluble components in the samples. Table 3 shows the results of the PAHs analysis. A total of 16 PAHs was identified.

**Table 1:** Concentration (mg/Kg) of elements in PM_2.5_

Element	Concentration	Element	Concentration
**Al**	2.09×10^3^	**As**	738
**Cr**	4.36	**Cu**	7.40
**Fe**	1.35×10^3^	**Mn**	42.4
**Ni**	0.934	**Pb**	8.25
**Sb**	16.9	**Zn**	4.08×10^3^
**Ca**	1.67×10^4^	**K**	1.94×10^3^
**Mg**	5.28×10^3^	**Na**	5.72×10^3^
**Si**	4.23×10^4^	**EC**	1.35×10^4^
**OC**	1.17×10^5^		

EC: element carbon; OC: organic carbon.

**Table 2:** Concentration (mg/Kg) of water soluble components in PM_2.5_

Element	Concentration	Element	Concentration
**Cl^−^**	552	**SO_4_^2−^**	1.25×10^4^
**NO_3_^−^**	386	**NH_4_^+^**	646

**Table 3:** Concentration (mg/Kg) of PAHs in PM_2.5_

Element	Concentration	Element	Concentration
**Phenanthrene**	0.47	**Anthracene**	0.014
**Fluoranthene**	1.43	**Pyrene**	1.28
**Benzo [a] anthracene**	16.9	**Chrysene**	0.818
**Benzo [a] pyrene**	0.463	**Benzo [k] fluoranthene**	0.164
**Benzo [b] fluoranthene**	0.157	**Dibenzo [a,h] anthracene**	0.088
**Benzo [g,h,i] perylene**	0.325	**Indeno [1,2,3-cd] pyrene**	0.10
**Naphthalene**	0.076	**Fluorene**	0.017

### Cognitive deficits in mice after PM_2.5_, FA or multi-pollutant exposure

**Figure 1:**
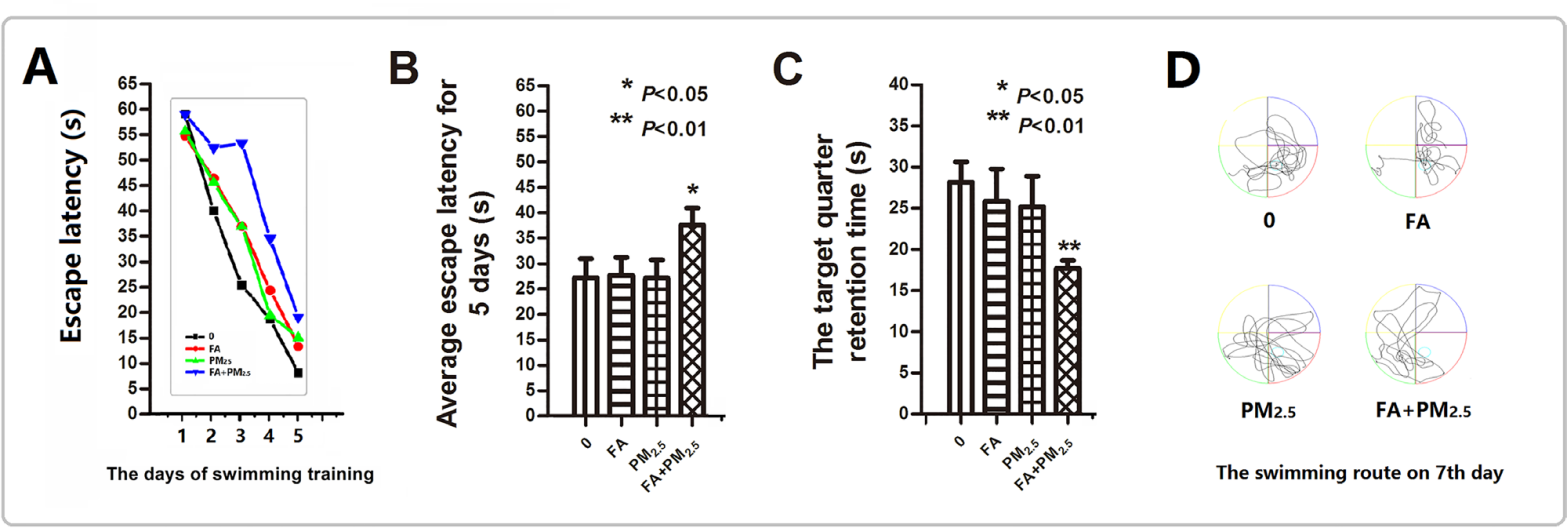
Morris water maze test after exposure **(A)** The escape latency for the first 5 days. **(B)** The average escape latency for the first 5 days. **(C)** The time spent swimming in the platform quadrant on the seventh day. **(D)** The swimming route on the seventh day. ^*^P<0.05, ^**^P<0.01, compared with the control group.

After 5 days of training, each of the exposure group mice exhibited a reduction in escape latency (EL) (Figure 1A). However, the mice that were exposed to multi-pollutant had significantly slower decrease. So the average escape latency for 5 days of co-exposure group was higher than control group (Figure 1B). On the seventh day, the time that mouse stayed in the platform quadrant (SE quadrant) was remarkably short for the mice that received co-exposure (Figure 1C). Looking at the swimming routes of the mice on the seventh day, it can be seen that the routes of the control, PM_2.5_, and FA groups were primarily in the platform quadrant, while the routes of co-exposure group were irregular and showed no purpose (Figure 1D).

### Effects of PM_2.5_, FA or multi-pollutant exposure on blood-brain barrier (BBB) permeability

**Figure 2:**
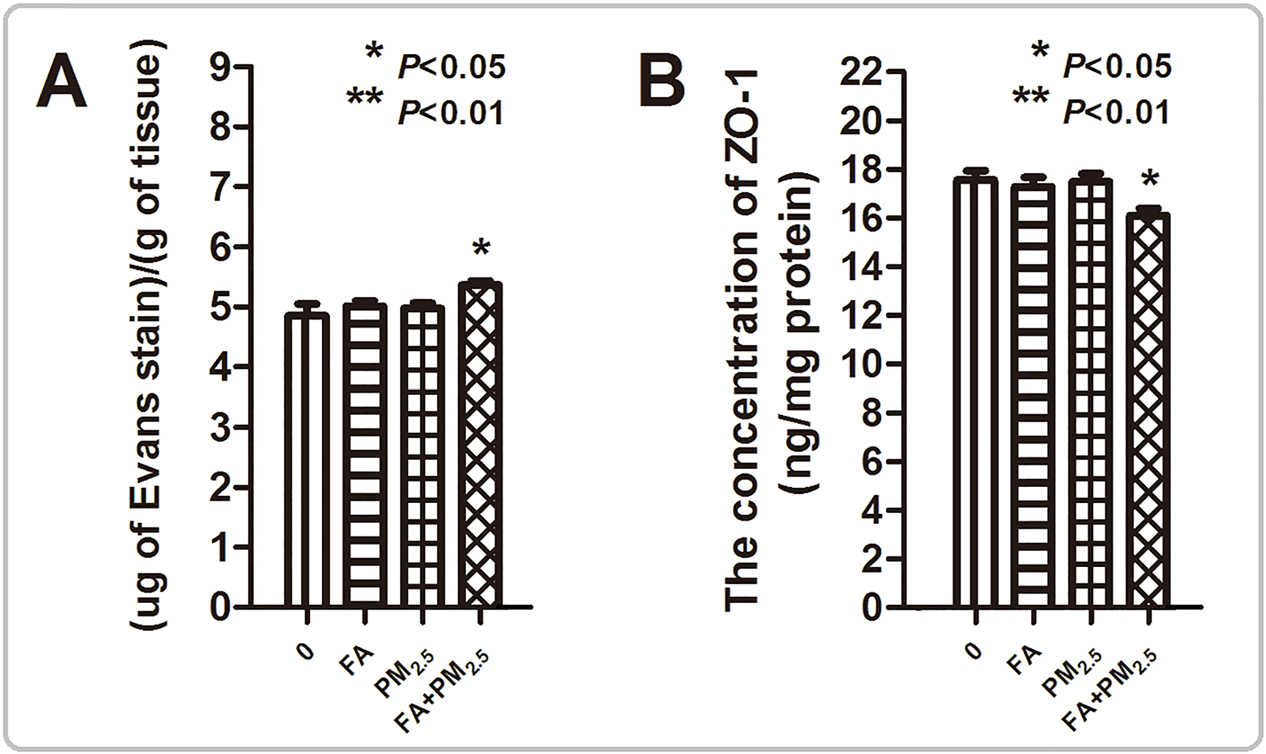
Evaluation of BBB permeability **(A)** Evans Blue Assay. **(B)** The concentration of ZO-1 in the mouse brain after exposure. ^*^P<0.05, ^**^P<0.01, compared with the control group.

An increase in Evans Blue (EB) stain accumulation was not seen in the brains of mice that were exposed to PM_2.5_ or FA alone, while accumulation was seen in the co-exposed mice. Depletion of Zonula occludens-1 (ZO-1) was also detected in the mice in the co-exposure group (Figure 2).

### Histopathological alterations of mice brains after PM_2.5_, FA or multi-pollutant exposure

**Figure 3:**
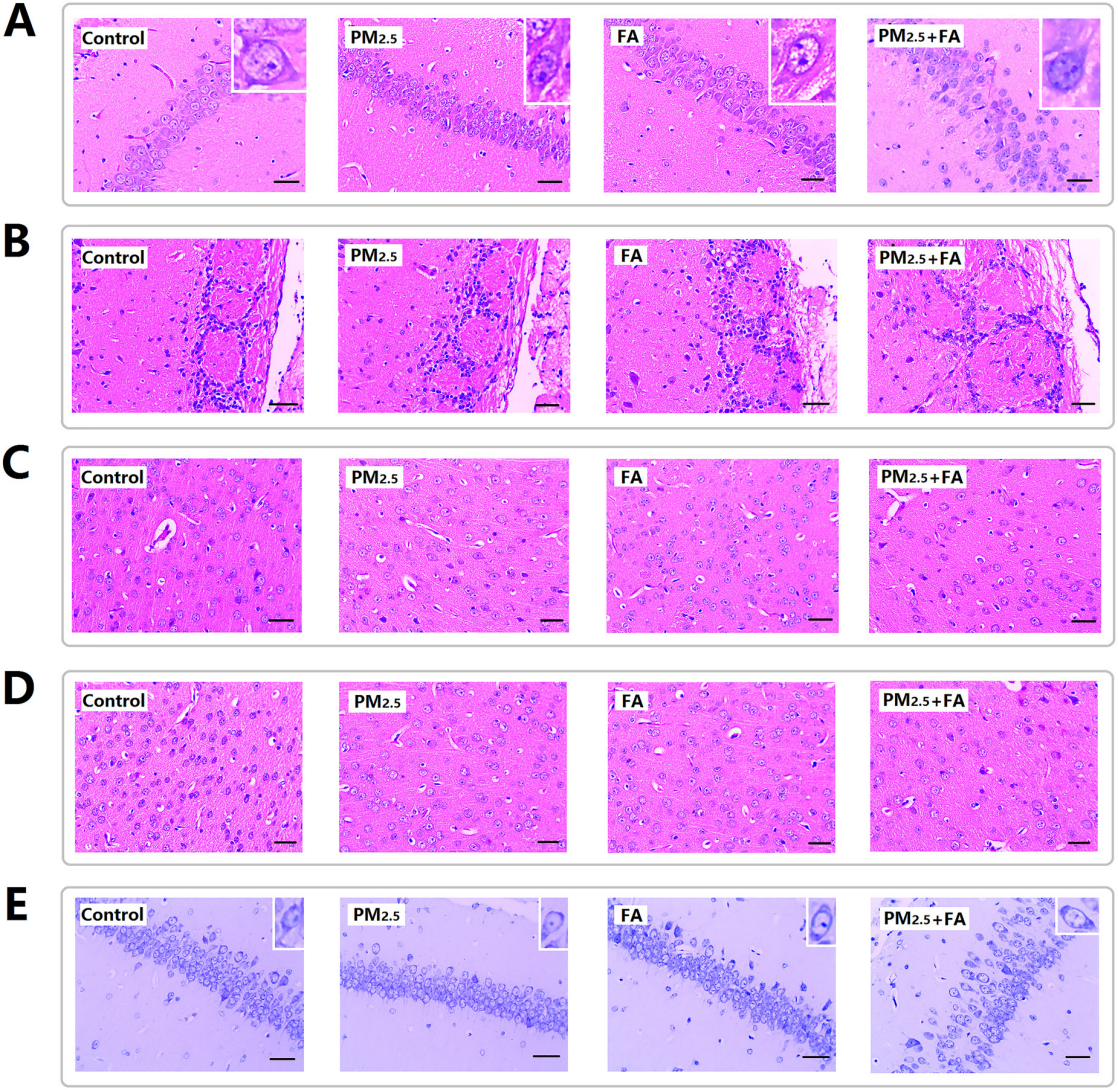
Representative images (×400) of the hippocampus, olfactory bulb, cerebral cortex and prefrontal cortex after exposure **(A)** H&E staining for hippocampus. **(B)** H&E staining for olfactory. **(C)** H&E staining for cerebral cortex. **(D)** H&E staining for prefrontal cortex. **(E)** Nissl staining for hippocampus. Scale=50μm.

After exposure, hippocampus, olfactory bulb, cerebral cortex and prefrontal cortex were examined. In Figure 3A the results showed that the pyramidal cells of the hippocampus CA_1_ region of control group are neatly aligned, the cells have clear edges are polygonal in shape, and the apical dendrites are clearly visible. Exposure to PM_2.5_ or FA induced zero or minor pathological alterations, while co-exposure induced cell arrangement loosening. Figure 3B shows the olfactory bulb changes after exposure, the structure of the synaptic glomerulus in the olfactory bulb of control group was integrated and arranged in a tidy manner. With co-exposure, the circular structure of the synaptic glomerulus was destroyed. Figure 3C & 3D shows histopathological changes in the cerebral cortex and prefrontal cortex after exposure. Compared with the control groups the cell morphology did not change significantly. However, co-exposure resulted in a decrease in cell numbers in the cerebral cortex and prefrontal cortex (Table 4).

**Table 4:** Cell number in cerebral cortex & prefrontal cortex

Group	Cerebral cortex		Prefrontal cortex	
	Cell number (Mean±SD)	*P* value	Cell number (Mean±SD)	*P* value
**0**	163.17±36.65		162.33±26.34	
**PM_2.5_**	152.33±19.43	0.5367	135.6±23.82	0.0329(^*^)
**FA**	165±22.23	0.9186	142.4±15.71	0.0586
**FA+ PM_2.5_**	119±19.9	0.0185(^*^)	118.33±17.48	0.0007(^**^)

^*^P<0.05, ^**^P<0.01, compared with the control group.

### Nissl substance decreased in the mouse brain after PM_2.5_, FA or multi-pollutant exposure

Nissl staining of the hippocampus after exposure is shown in Figure 3E, and clearly demonstrates the normal morphology and clear cytoplasmic staining of cells in control animals, and the down-regulation of nissl substance was did by co-exposure (Table 5).

**Table 5:** The average optical density (OD) of Nissl staining

Group	Nissl staining	
	OD (Mean±SD)	*P* value
**0**	0.777±0.030	
**PM_2.5_**	0.787±0.041	0.664
**FA**	0.818±0.043	0.1174
**FA+ PM_2.5_**	0.678±0.037	0.0019(^**^)

^**^P<0.01, compared with the control group.

### Exposure to PM_2.5_, FA or multi-pollutant increased beta-amyloid plaques 1-42 (Aβ_1-42_) and hyper-phosphorylated tau (Tau-P) levels in mice brains

**Figure 4:**
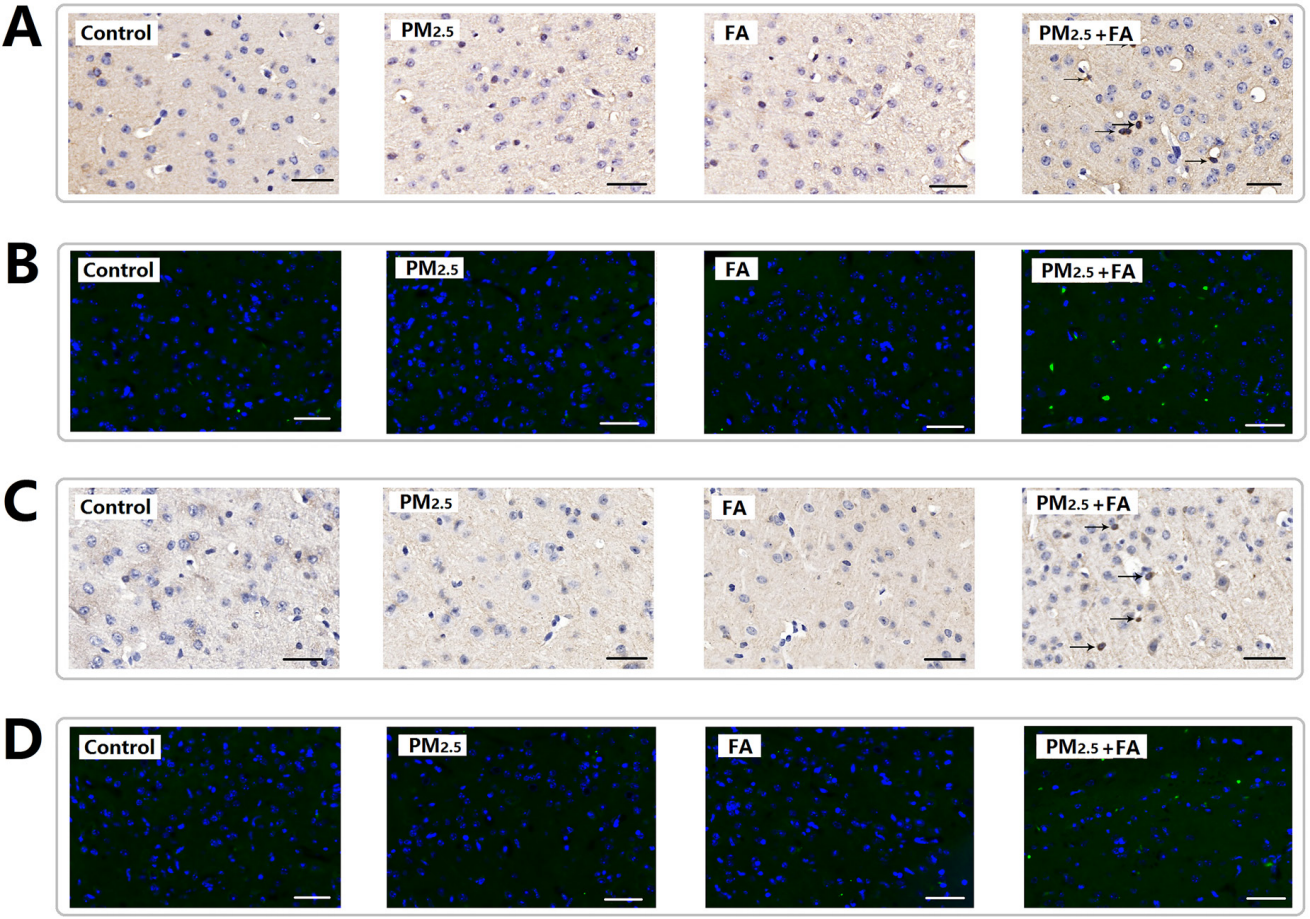
Representative images (×400) of the expression of Aβ1-42 and Tau-P as determined by immune-histochemical staining (brown color stain) and immunofluorescence **(A)** Expression of Aβ_1-42_ (HIS). **(B)** Expression of Aβ_1-42_ (IF, green). **(C)** Expression of Tau-P (HIS). **(D)** Expression of Tau-P (IF, green). Nuclei were stained by DAPI reagents (blue). Scale=50μm.

After exposure, neuronal accumulation of Aβ_1-42_ was found to be present in the cerebral cortex of co-exposure group (Figure 4A & 4B), PM_2.5_ or FA exposure alone did not change the expression of Aβ_1-42_ (Table 6). The expression of Tau-P in the cerebral cortex (Figure 4C & 4D) after exposure was very similar to Aβ_1-42_. PM_2.5_ or FA exposure alone did not change the expression of Tau-P, while after co-exposure, the expression of Tau-P in the cerebral cortex was observed to increase significantly (Table 6).

**Table 6:** The average optical density (OD) of Aβ_1-42_, Tau-P, Iba1, GFAP immunohistochemical staining

Group	Aβ_1-42_		Tau-P	
	OD (Mean±SD)	*P* value	OD (Mean±SD)	*P* value
**0**	0.0018±1.520E^−4^		0.0060±0.0012	
**PM_2.5_**	0.0021±2.746E^−4^	0.0726	0.0063±0.0021	0.8234
**FA**	0.0017±2.547E^−4^	0.4407	0.0063±9.339E^−4^	0.7455
**FA+ PM_2.5_**	0.0190±0.0021	<0.0001(^**^)	0.0080±9.368E^−4^	0.0189(^*^)
**Group**	**Iba1**		**GFAP**	
	**OD (Mean±SD)**	***P* value**	**OD (Mean±SD)**	***P* value**
**0**	0.0010±4.001E^−4^		0.0502±0.0107	
**PM_2.5_**	0.0010±2.251E^−4^	0.8761	0.0538±0.0103	0.6035
**FA**	0.0011±4.907E^−4^	0.6101	0.0543±0.0100	0.5464
**FA+ PM_2.5_**	0.0108±0.0048	0.0018(^**^)	0.0633±0.0054	0.0405(^*^)

^*^P<0.05, ^**^P<0.01, compared with the control group.

### Glia activation in the mouse brain after PM_2.5_, FA or multi-pollutant treatment

**Figure 5:**
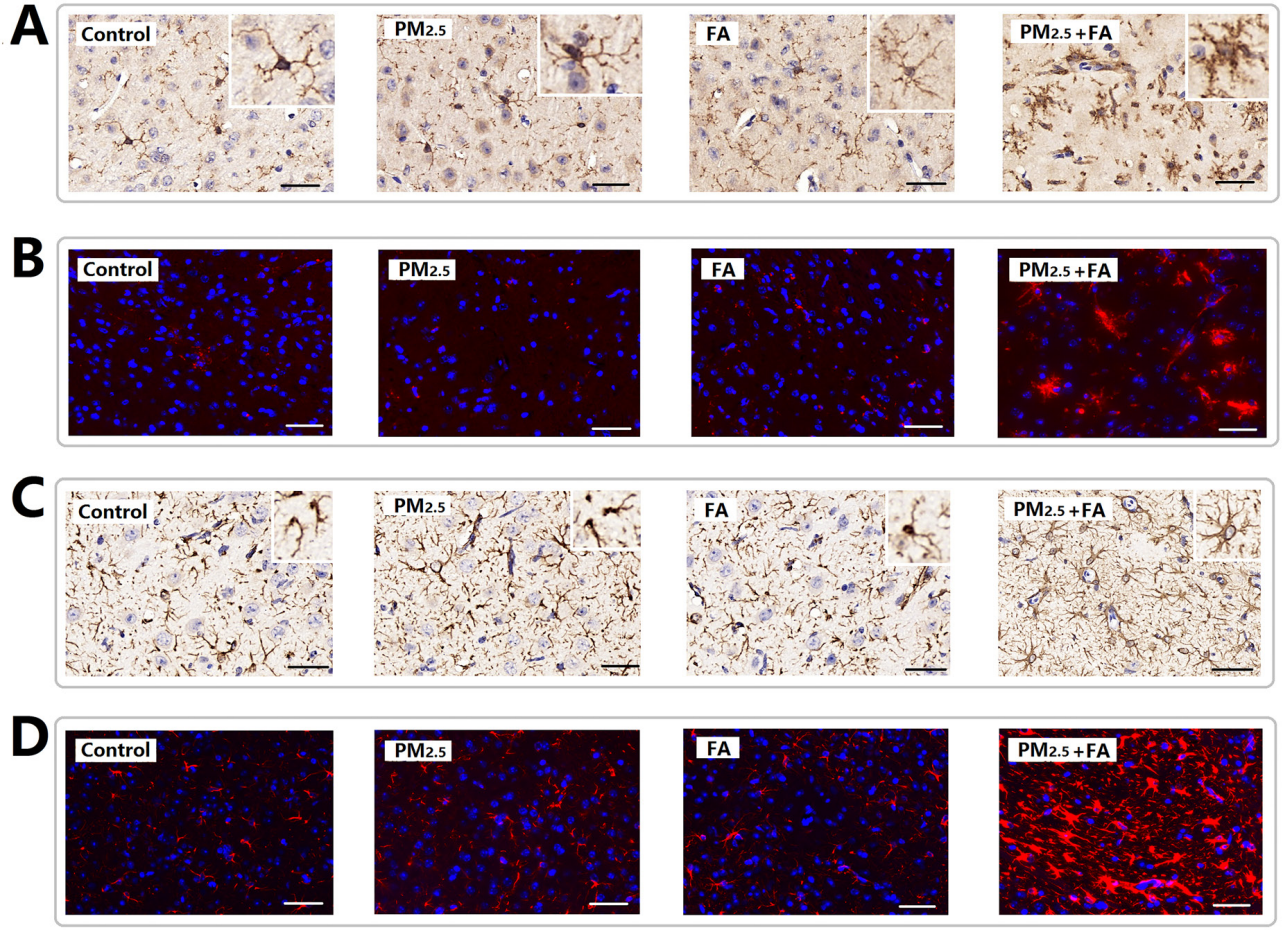
Representative images (×400) of the expression of Iba1 and GFAP as determined by immune-histochemical staining (brown color stain) and immunofluorescence **(A)** Expression of Iba1 (HIS). **(B)** Expression of Iba1 (IF, red). **(C)** Expression of GFAP (HIS). (D) Expression of GFAP (IF, red). Nuclei were stained by DAPI reagents (blue). Scale=50μm.

As Figure 5A & 5B shows, in the control animals the microglia display a ramified morphology with small cell bodies and elongated cell neurites, all characteristic features of resting microglia. After co-exposure, Iba1-positive cell size increases, neurites retract and coarsen, and some spines were found on the neurites. Significant changes in Iba1 levels were observed (Table 6).

Through Figure 5C & 5D, astrocyte activation can also be observed. In vehicle animals, the astrocytes presented their normal morphology with non hypertrophic cell bodies and a ramified pattern of their branches. After co-exposure, astrocytes were activated, this activation was characterized by increased cell body volume and branches became more ramified. In addition, expression of fibrillary acidic protein (GFAP) in the cerebral cortex increased significantly (Table 6). However, PM_2.5_ or FA exposure alone did not induce microglia and astrocytes activation significantly.

### PM_2.5_, FA or multi-pollutant exposure exacerbates the oxidative stress (OS) and inflammation level in the mouse brain

**Figure 6:**
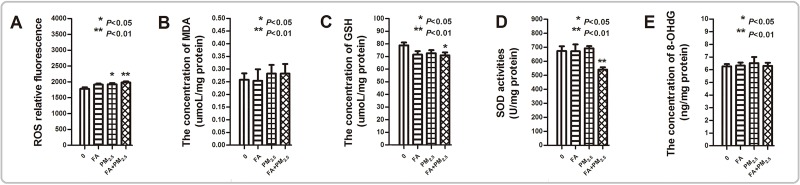
The oxidative stress level after PM_2.5_, FA or multi-pollutant exposure **(A)** The relative fluorescence of ROS in the mouse brain after exposure. **(B)** The concentration of MDA in the mouse brain after exposure. **(C)** The concentration of GSH in the mouse brain after exposure. **(D)** The SOD activity in the mouse brain after exposure. **(E)** The level of 8-OH-dG in the mouse brain after exposure. ^*^P<0.05, ^**^P<0.01, compared with the control group.

As Figure 6 shows, exposure to PM_2.5_ or FA alone had no or only minor adverse effects on the levels of OS in the mouse brain. But co-exposure exacerbated OS. Significant increases in reactive oxygen species (ROS) levels (Figure 6A), and a marked decrease in glutathione (GSH) content and superoxide dismutase (SOD) activity were seen after co-exposure (Figure 6C & 6D). However, co-exposure not changed the levels of malondialdehyde (MDA) and 8-hydroxy-2-deoxyguanosine (8-OH-dG).

**Figure 7:**
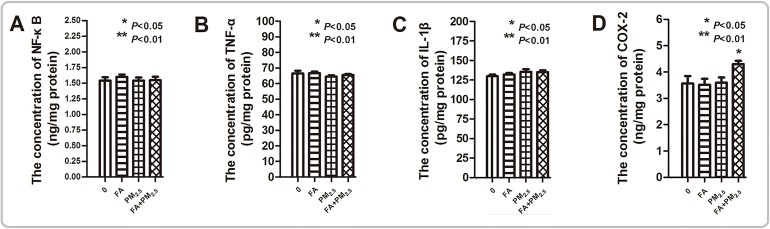
The inflammation level after PM_2.5_, FA or multi-pollutant exposure **(A)** The concentration of NF-κB in the mouse brain after exposure. **(B)** The concentration of TNF-α in the mouse brain after exposure. **(C)** The concentration of IL-1β in the mouse brain after exposure. **(D)** The concentration of COX-2 in the mouse brain after exposure. ^*^P<0.05, ^**^P<0.01, compared with the control group.

The expression levels of nuclear factor κB (NF-κB), tumor necrosis factor α (TNF-α), interleukin-1β (IL-1β), and Cyclooxygenase 2 (COX-2) were measured to reflect the inflammation level in the mouse brain (Figure 7). PM_2.5_ or FA exposure alone did not change these cytokines expression. In the co-exposure group, the synergistic effect of co-exposure on COX-2 expression was significant.

## DISCUSSION

People usually consider that when we exposure to certain pollutant at its hygienic standard even slightly higher than the standard has zero or minor adverse effects. Actually, we may have a mistake. In fact, environmental toxin exposure is a multi-pollutants co-exposure system. The synergistic effects of pollutants co-exposure may lead to unexpected results.

This study investigated the impact of exposure to PM_2.5_ or FA alone at hygienic standard or actually daily exposure level, as well as exposure to a mixture of PM_2.5_ plus FA on AD-like pathology in experimental animals. The results confirmed that PM_2.5_ or FA exposure alone had little or no effect on the mouse brain, however, co-exposure to PM_2.5_ plus FA had a significant synergistic adverse effect.

Acquisition and maintenance of spatial memory after exposure was assessed using a MWM to observe any cognitive function decline. The test indicated that in this study although exposure to PM_2.5_ or FA alone had no adverse effects on mice cognitive function, the co-exposure to PM_2.5_ plus FA induced significant cognitive decline. To explore the reasons underlying these adverse effects, this study investigated the possibility of damage to the internal brain structure. Some epidemiological and toxicological studies have confirmed that air pollution induced brain tissue damage in areas such as the BBB, the hippocampus, the olfactory bulb and the cortex, is similar to pathological symptoms of AD [10].

The results showed that after co-exposure, the permeability of the BBB deteriorated, structural changes in the hippocampus, olfactory bulb, cerebral cortex and prefrontal cortex were investigated. Hippocampus plays a critical role in spatial memory formation [11], and olfactory dysfunction also has a positive correlation with the risk of increasing cognitive decline [12, 13]. The histological observations of the hippocampus and olfactory bulb demonstrated that PM_2.5_ plus FA co-exposure induced pyramidal neurons of the hippocampus to be damaged, and the destruction of the structure of olfactory bulb. Additionally, chromatolysis (Nissl substance loss) in hippocampus and cell numbers decreased in cerebral and prefrontal cortex was demonstrated after co-exposure. All these type of damage is a common pathological phenomenon in neurodegenerative diseases, and may affect neural signal transmission and memory formation, eventually contributing to cognitive function decline.

Abnormally high expression of Aβ_1-42_ and Tau-P destroy the stability of microtubules and axonal transport, eventually causing neuronal death, and inducing the occurrence of neurodegenerative diseases [14–16]. In this research, Aβ_1-42_ and Tau-P expression were induced by co-exposure. The abnormal expression of these proteins may be responsible for a decrease in neuron cell number in the cerebral cortex.

In order to explore the molecular mechanisms which lead to brain damage after exposure, some key upstream events were detected. According to some studies, OS and inflammation are part of the pathology of AD. One plausible mechanism is that OS and inflammation modifies the aggregation and fibrillation rate of proteins, and induces tau phosphorylation, potentially affecting AD etiology [6, 17, 18]. According to the results, the synergistic effect of co-exposure was detected in ROS accumulation, and GSH depletion and SOD activity decline. Following the hierarchical OS hypothesis, the effect of OS depends upon its degree. A high level OS will cause pro-inflammatory effects associated with diverse cellular activities [19]. This study showed the synergistic effect of co-exposure was reflected in the expression of COX-2.

In addition to understanding how ROS and cytokines accumulate after exposure, this study addressed some cell types that may be responsible for this. Hyperactive microglia can be found in response to endogenous disease proteins, inflammation cytokines, neuronal death, and environmental toxins [20]. ROS derived from activated microglia are crucial for neurotoxicity induced by toxins. Excess ROS re-induces inflammation, cell damage and disease protein accumulation [6]. The cycle of microglia activation causing accumulation of ROS results in neurotoxicity or the occurrence of disease. Astrocytes are essential abundant cells in the CNS, and are important for BBB integrity, the formation of synapses, the homeostasis of neurotransmitters, etc [6]. Activated astrocytes highly up-regulate many classical complementary cascade genes previously shown to be destructive to synapses, and enhance the expression of some cytokines which can form a glial scar [21, 22]. In this study, Iba1 and GFAP were chosen as important markers of microglial and astrocyte activation. Consistent with the above results, after co-exposure, microglia and astrocytes were activated. While exposure to PM_2.5_ or FA alone did not enhance the expression of Iba1 and GFAP, the synergistic effect of co-exposure was significant. The increasingly activated microglia and astrocytes might be the main source of excess ROS and cytokines.

According to the results in this study, PM_2.5_ or FA alone at hygienic standard or actually daily exposure level had little or no damage on mice brain, while the co-exposure induced cognitive deficits, BBB damage, brain pathological alterations, Aβ_1-42_ and Tau-P accumulation, glia activation, OS and inflammation in mouse brain, which are common pathological phenomenon in AD.

**Figure 8:**
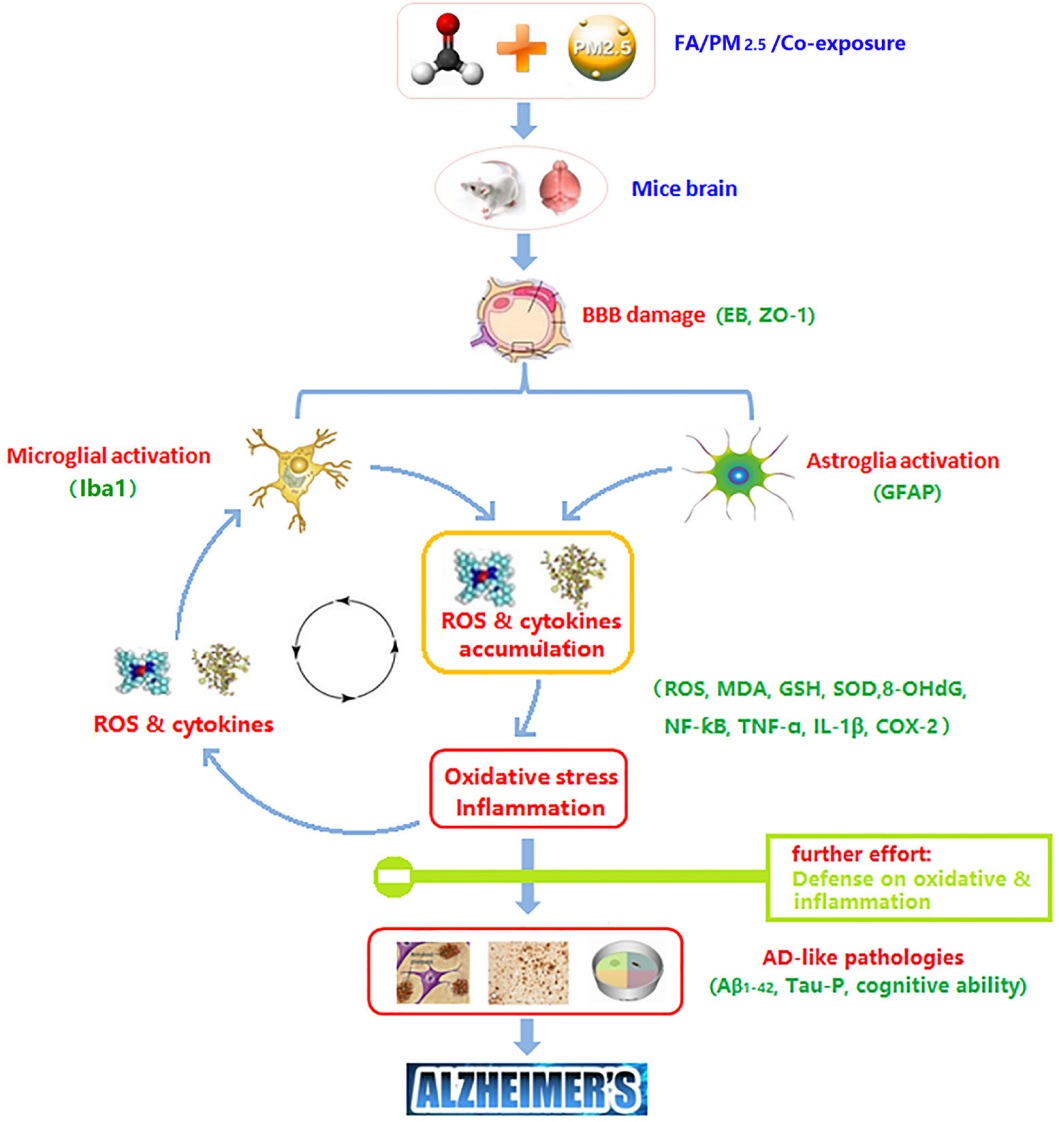
Potential mechanism of PM_2.5_, FA or multi-pollutant exposure induced damage in the mouse brain Figure 8 was originally made by Xudong Liu.

In conclusion, this work suggests that one week of exposure to PM_2.5_ or FA alone at hygienic standard or actually daily exposure level resulted in little or no damage. However, mice are more likely to reflect AD-like pathologies after exposure to a combination of PM_2.5_ plus FA at these levels. These safe levels for alone exposure turned into dangerous at co-exposure. So, whether you still think that you are safe to expose pollutants at hygienic standards or actually daily exposure levels? You may have already received the injury through pollutants co-exposure. In the future, take synergistic effects of pollutants co-exposure into the process of making hygienic standard, the maximum allowable concentration of each pollutant may be lower. And further effort in prevention studies, such as how to defend against OS (eg. antioxidants) and inflammation (eg. nsaids) after exposure was stimulated (Figure 8).

## MATERIALS AND METHODS

### Animals

Specified pathogen-free class (SPF) Male C57BL/6J mice (6 weeks old, 22±2g) were supplied by the Hubei Province Experimental Animal Center (Wuhan, China). All mice were maintained in pathogen-free cages at 20-25°C temperature with 50-70% humidity and a 12 h light/dark cycle. All mice were provided with a commercial diet and filtered water *ad libitum*.

All experimental procedures were approved by Office of Scientific Research Management of Central Normal University (Wuhan, China). Animal experiments were conducted under the National Institutes of Health Guide for the Care and Use of Laboratory Animals, with a certificate of Application for the Use of Animals dated 20 December, 2015 (approval ID: CCNU-IACUC-2016-003).

### Collection of PM_2.5_

A high traffic, total suspended particulates (TSP), sampler (KC-1000, Qingdao) was used to collect ambient air composed of PM<2.5μm (fine/ultrafine; F/UF) in Wuhan, China. The collected PM_2.5_ glass filter membranes were cut into small pieces, processed ultrasonically in ultrapure water for 40 min, after which the filter membranes were thrown away. The extracted liquid was vacuum freeze dried and cryogenically preserved. Before the experiment, the freeze-dried particulate matter was mixed with normal saline to get a particle suspension, which was then subjected to ultrasonic oscillation for 15 min, ensuring that the suspension was uniform and sterile.

### Analysis of components in the PM_2.5_

The elements in the samples were analyzed by inductively coupled plasma atomic emission spectroscopy (ICP-AES, 61E Trace and ICP-750, Thermo Jarrell-Ash, MA) after acid digestion with mixed acids (68% nitric, 38% hydrofluoric, and 70% perchloric = 5:1:1) was performed at 180°C for 3 h. The water soluble components in the samples were analyzed using an ion chromatograph (DX-100, Dionex, Sunnyvale, CA) and ICP-AES (61E Trace, Thermo Jarrell-Ash). Polycyclic Aromatic Hydrocarbons (PAHs) were analyzed according to a previously reported method [23].

### Preparation of exposure pollutants

### Preparation of FA

According to the “Hygienic standard for formaldehyde in the indoor air of a house (GB/T 16127-1995)” in China, the upper limit for an acceptable concentration of FA in indoor air is 0.08 mg/m^3^. The tidal volume of a human is roughly 15 m^3^/day [24], so the exposure dose of FA at the maximum permissible concentration is 0.08 mg/m^3^×15m^3^/day =1.2 mg/day. By converting the exposure dosage to mice [25], so that the exposure dosage for mice is equivalent to that experienced by humans, got 0.155 mg/Kg/day. The exposure FA is prepared from formalin and the volume of intranasal instillation is 10 μL.

### Preparation of PM_2.5_

It is a pity that there is no hygienic standard for indoor PM_2.5_ in China. According to some studies, for example, in Beijing, Shanghai, Wuhan, Chongqin and Nanchang, the indoor PM_2.5_ concentrations were measured at 85, 142, 92, 211, 103 μg/m^3^ during the test period, respectively [25]. Using these measured concentrations as a guide, 100 μg/m^3^ were used as the exposure dosage in this study. Converting this exposure dosage to mice results in an exposure of 0.193 mg/Kg/day for the mice. The exposure PM_2.5_ is dissolved in normal saline and the volume of intranasal instillation is 10 μL.

### Preparation of the multi-pollutant

In this study, the co-exposure dosage of the multi-pollutant is 0.155 mg/Kg/day FA plus 0.193 mg/Kg/day PM_2.5_. The volume of intranasal instillation is 10 μL.

### Experimental protocol

**Figure 9:**
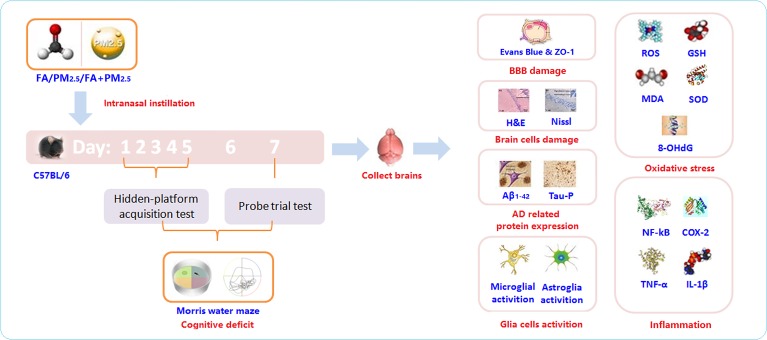
The experimental protocol

Mice divided randomly into 4 groups, were exposed (intranasal instillation) daily to 0, PM_2.5_, FA or the multi-pollutant for 7 days. Throughout the exposure period, the Morris Water Maze (MWM) was performed to evaluate the cognitive ability of the mice after exposure. After the MWM, some pathological hallmarks of AD and some key upstream events were investigated (Figure 9).

### Morris water maze (MWM)

Acquisition and maintenance of spatial memory by the trained rats was assessed by using a MWM test as described previously [26, 27]. The maze was a 100 cm diameter pool, filled with water to a depth of 20 cm. The water was kept at room temperature. The pool was divided into four quadrants (N, E, S, W quadrants) and the platform was placed 1 cm below the water surface, in the center of the SE quadrant. A camera recorded the tracks of mice over the pool. In this study, the training continued from the first to the fifth day. The mice were put into the pool from the center of the NE, NW and SW quadrants, each mouse trained three times each day. The time that the mice spend looking for the platform is termed “EL”. EL was measured to reflect the spatial memory acquisition ability of mice. If a mouse failed to find the platform within 60s, the EL was recorded as 60s. Then guided the mouse to the platform and kept it there for 30s so it could learn (hidden-platform acquisition test for the first five days). On the sixth day, no MWM was performed (forgetting-period), but on the seventh day the MWM was performed again, but with no platform in the pool. In this case, the mice were also put into the pool from the center of the NE, NW and SW quadrants and forced to swim for 60s. During this period, the swimming tracks were recorded by camera. The swimming time spent in the SE quadrant (where the platform was previously) reflects the spatial memory ability of the mice after exposure (probe trial test on the seventh day).

### Evaluation of BBB permeability

24 hours after the final intranasal instillation, a 0.5% solution of EB in normal saline was injected intravenously. The stain was allowed to circulate for 1 hour, after which the mouse brain was collected, and the EB content in the mouse brain was measured as previously described [28]. An ELISA kit (BlueGene Biotech Company, Shanghai, China) was used to measure the ZO-1 concentration in the brain. The kit was used according to the manufacturer's instructions.

### Histological examination

Brain samples were sectioned into slices for hematoxylin and eosin (H&E)- and nissl-staining as previously described [29]. The number of cells in each of the slices from the cerebral cortex and the prefrontal cortex were counted, and the average optical density (OD) of each nissl-stained slice was tested by software (Image-Pro Plus 6.0, Media Cybernetics, Bethesda, MD, USA).

### Immunohistochemistry for GFAP, Iba1, Aβ_1-42_ and Tau-P

Brain sections were incubated with diluted primary antibodies (rabbit anti-GFAP, Iba1, Aβ_1-42_, Tau-P antibody, 1:200 dilution). Then antibody binding was detected by biotinylated immunoglobulins and avidin-biotin peroxidase complex as previously described [30]. Immunostained sections were observed under a DM 4000B microscope. Image-Pro Plus 6.0 sofware was used to evaluate the staining intensity OD.

### Immunofluorescence for GFAP, Iba1, Aβ_1-42_ and Tau-P

Brain sections were blocked with 5% bovine serum albumin (BSA) for 30 min and incubated with anti-paxillin antibody or anti-phosphotyrosine antibody at 4°C overnight. Subsequently, brain sections were washed and incubated with Cy-3-conjugated or FITC-conjugated secondary antibody at 37°C for 60 min, washed again, mounted, and examined under a fluorescence microscope. The cells were also counterstained with DAPI for nuclear staining. Fluorescence images were captured using a Leica Qwin V3 (Leica Microsystems).

### Brain sample homogenate

Mice brains were collected and weighed. The brain sample homogenate was made as previously described [28].

### Assessment of ROS, MDA and GSH

The ROS, MDA and GSH content of the mouse brain was measured using 2’,7’-dichlorofluorescin diacetate (DCFH-DA), 2-thiobarbituric acid (TBA), and 3-carboxy-4-nitrophenyl disulfide (DTNB) respectively, as previously described [28].

### Analysis of SOD activity

SOD activity was determined using a SOD assay kit (Jiancheng, Nanjing, Jiangsu, China). The kit was used following instructions provided by the manufacturer.

### Detection of 8-OH-dG content

An ELISA kit (R&D System, Minneapolis, MN, USA) was used to measure the 8-OH-dG concentration in the brain supernatant according to the manufacturer's instructions. The sensitivities of the ELISA kit was 0.1 ng/mL.

### Analysis of NF-κB, TNF-α, IL-1β and COX-2 content

ELISA kits provided by eBioscience (eBioscience, San Diego, CA, USA) were used to measure the concentrations of NF-κB, TNF-α, IL-1β. And kit supplied through BlueGene (BlueGene Biotech Company, Shanghai, China) was used to detect the content of COX-2. All kits were used according to the directions provided by the manufacturers The sensitivities of the ELISA kits were 0.1 ng/mL for NF-κB and COX-2, 8 pg/mL for TNF-α, and 80 pg/mL for IL-1β.

### Statistical analyses

Data were presented as the mean ± standard error (SE). Repeated ANOVAs combined with a post hoc Tukey test were used for the first 5 day's average escape latency analyses in the MWM to determine whether there was any significant difference between groups. All other data collected were analyzed by a one-way ANOVA followed by a Tukey test. Values of *p*<0.05 were considered statistically significant. Data analyses were carried out using SPSS 13.0 (Chicago, IL, USA) and generated statistical graphs using GraphPad Prism 5.0 (San Diego, CA, USA).

## Abbreviations

AD: Alzheimer's disease
PM_2.5_: ambient fine particles
FA: formaldehyde
Aβ: beta-amyloid plaques
Tau-P: hyper-phosphorylated tau
PM: particulate matter
SPF: specified pathogen-free
EC: element carbon
OC: organic carbon
TSP: total suspended particulates
F/UF: fine/ultrafine
MWM: Morris water maze
NE: northeast
SE: southeast
NW: northwest
SW: southwest
EL: escape latency
BBB: blood-brain barrier
EB: Evans Blue
ZO-1: Zonula occludens-1
Aβ_1-42_: beta-amyloid plaques 1-42
Tau-P: hyper-phosphorylated tau
H&E: hematoxylin and eosin
OS: oxidative stress
OD: average optical density
GFAP: glial fibrillary acidic protein
DAB: diaminobenzidine
PBS: phosphate-buffered saline
ROS: reactive oxygen species
MDA: malondialdehyde
GSH: glutathione
DCFH-DA: 2′, 7′-dichlorofluorescin diacetate
DTNB: 3-carboxy-4-nitrophenyl disulfide
SOD: superoxide dismutase
8-OH-dG: 8-hydroxy-2-deoxyguanosine
COX-2: cyclooxygense 2
NF-κB: nuclear factor κB
TNF-α: tumor necrosis factor α
IL-1β: interleukin-1β
SE: standard error
